# Tat-exported peptidoglycan amidase-dependent cell division contributes to *Salmonella* Typhimurium fitness in the inflamed gut

**DOI:** 10.1371/journal.ppat.1007391

**Published:** 2018-10-31

**Authors:** Mayuka Fujimoto, Ryosuke Goto, Riku Hirota, Masahiro Ito, Takeshi Haneda, Nobuhiko Okada, Tsuyoshi Miki

**Affiliations:** Department of Microbiology, School of Pharmacy, Kitasato University, Tokyo, Japan; Emory University School of Medicine, UNITED STATES

## Abstract

*Salmonella enterica* serovar Typhimurium (*S*. Tm) is a cause of food poisoning accompanied with gut inflammation. Although mucosal inflammation is generally thought to be protective against bacterial infection, *S*. Tm exploits the inflammation to compete with commensal microbiota, thereby growing up to high densities in the gut lumen and colonizing the gut continuously at high levels. However, the molecular mechanisms underlying the beneficial effect of gut inflammation on *S*. Tm competitive growth are poorly understood. Notably, the twin-arginine translocation (Tat) system, which enables the transport of folded proteins outside bacterial cytoplasm, is well conserved among many bacterial pathogens, with Tat substrates including virulence factors and virulence-associated proteins. Here, we show that Tat and Tat-exported peptidoglycan amidase, AmiA- and AmiC-dependent cell division contributes to *S*. Tm competitive fitness advantage in the inflamed gut. *S*. Tm *tatC* or *amiA amiC* mutants feature a gut colonization defect, wherein they display a chain form of cells. The chains are attributable to a cell division defect of these mutants and occur in inflamed but not in normal gut. We demonstrate that attenuated resistance to bile acids confers the colonization defect on the *S*. Tm *amiA amiC* mutant. In particular, *S*. Tm cell chains are highly sensitive to bile acids as compared to single or paired cells. Furthermore, we show that growth media containing high concentrations of NaCl and sublethal concentrations of antimicrobial peptides induce the *S*. Tm *amiA amiC* mutant chain form, suggesting that gut luminal conditions such as high osmolarity and the presence of antimicrobial peptides impose AmiA- and AmiC-dependent cell division on *S*. Tm. Together, our data indicate that Tat and the Tat-exported amidases, AmiA and AmiC, are required for *S*. Tm luminal fitness in the inflamed gut, suggesting that these proteins might comprise effective targets for novel antibacterial agents against infectious diarrhea.

## Author summary

For proteins residing outside the bacterial cytoplasm, transport is an essential step for adequate function. The twin-arginine translocation (Tat) system enables the transport of folded proteins across the cytoplasmic membrane in prokaryotes. It has recently become clear that this system plays a pivotal role in the detrimental effects of many bacterial pathogens, suggesting Tat as a novel therapeutic target against their infection. In particular, the bacterial enteropathogen *Salmonella* Typhimurium causes foodborne diarrhea by colonizing the gut interior space. Here, we describe that the *S*. Typhimurium Tat system contributes to intestinal infection by facilitating colonization of the gut by this pathogen. We also identify that two Tat-exported enzymes, peptidoglycan amidase AmiA and AmiC, are responsible for the Tat-dependent colonization. *S*. Typhimurium strains having nonfunctional Tat systems or lacking these enzymes undergo filamentous growth in the gut interior owing to defective cell division. Notably, this chain form of *S*. Typhimurium cells is highly sensitive to bile acids, rendering it less competitive with native bacteria in the gut. The data presented here suggest that the Tat system and associated amidases may comprise promising therapeutic targets for *Salmonella* diarrhea, and that controlling bacterial shape might be new strategy for regulating intestinal enteropathogen infection.

## Introduction

Protein translocation constitutes an essential cell function in all types of prokaryotic or eukaryotic cells. Accordingly, bacteria have evolved several sophisticated translocation systems to transport proteins into, or across, the cytoplasmic membrane. In particular, for bacterial pathogens, this process is suspected to contribute to pathogenesis and interbacterial competition because the substrates include virulence factors in certain cases. Most general protein transport occurs via the Sec system, which predominantly transports unfolded proteins across the cytoplasmic membrane [1]. In addition, other additional transport systems have also evolved to facilitate the transport of different types of protein.

The twin-arginine translocation (Tat) system is essential for the appropriate localization of a number of folded proteins that function outside the cytoplasm [2]. In most Gram-negative bacteria including *Salmonella*, the Tat transport machinery is composed of the integral membrane proteins TatA, TatB, and TatC, which are inserted in the inner membrane [3, 4]. These proteins form their respective homo-oligomers in the membrane, with TatB and TatC subsequently associating as a complex (TatBC) [5] whereas TatA remains separate. During the initiation of protein export, a folded substrate protein docks at the TatBC complex through binding of its twin arginine signal peptide [6]. TatA tetramer is then recruited to the substrate-bound TatBC complex to form the translocation channel [7, 8]. Finally, the substrate is transported by crossing the membrane via the polymerized TatA component, whereupon its signal sequence is cleaved and the TatA proteins disassociate [9–11]. Notably, the Tat system is conserved among many bacterial pathogens [3, 12], with Tat substrates including both virulence factors and virulence-associated proteins [13–24]. Hence, it is well recognized that the Tat system is implicated in bacterial virulence [4, 25], leading to the hypothesis that the Tat system may constitute a novel therapeutic target against infection with bacterial pathogens.

*Salmonella enterica* serovar Typhimurium (*S*. Tm) is a common cause of diarrhea worldwide [26]. Following oral infection and upon reaching the gut lumen, *S*. Tm relies on flagella-based motility to gain access to the epithelial surface of the intestine [27, 28]. During its interaction with the host mucosa, genomic islands, termed SPI-1 and SPI-2, encoding type III secretion systems (ttss-1 and ttss-2) allow the bacterium to invade the epithelial cells and elicit mucosal inflammation [29, 30]. This inflammation depends upon activation of the NAIP/NLRC4 inflammasome, resulting in secretion of the inflammatory cytokines IL-1β and IL-18 [31–33]. During the initial stages (the first 12–18 hours post-infection), gut inflammation acts as an innate defense by reducing *S*. Tm loads in the infected tissue [32]. However, at later stages, *S*. Tm can instead benefit from gut inflammation for its own competitive growth advantage in the gut lumen [34]. Accumulating evidence has recently supported several underlying mechanisms for explaining the inflammation-mediated outgrowth blooming of *S*. Tm (reviewed in [28, 35, 36]). For example, gut inflammation provides electron acceptors, which promote anaerobic respiration of *S*. Tm [37]. Inflammation-based transmigration of polymorphonuclear leukocytes into the gut lumen leads to the generation of tetrathionate (S_4_O_6_^2–^) via reactive oxygen species. *S*. Tm, but not gut commensal bacteria, can use the tetrathionate as a terminal electron acceptor for anaerobic respiration, and thereby out-grow the competing microbiota in the inflamed gut. Although these findings provide an initial understanding of the beneficial competitive growth of *S*. Tm in the inflamed gut, a complete comprehension of the molecular mechanisms underlying this bloom remains to be elucidated.

Previous studies have shown that the Tat system is involved in *Salmonella* virulence [38–40]. For example, an *S*. Enteritidis mutant strain lacking a functional Tat system exhibits pleiotropic defects in virulence, with attenuated invasiveness into cultured cells, colonization of the cecum, and systemic infection in chickens [38]. Furthermore, the *tatC* mutant of *S*. Tm has been shown to be attenuated in a mouse model for typhoid fever in humans [39, 40]. This is also suspected to be attributable to multiple defects including attenuated invasion of macrophages, resistance to antimicrobials, motility, and expression of ttss-1 and ttss-2. However, the classical mouse model for typhoid fever was employed to evaluate virulence in these previous studies [39, 40], in which orally infected *S*. Tm causes systemic infection such as typhoid fever without gastrointestinal disease. The absence of enterocolitis is explained by the fact that the *S*. Tm strains cannot colonize the gut continuously owing to the competing gut microbiota. Therefore, it is completely unknown whether the Tat system is involved in *Salmonella* gut infection.

Alternatively, in the present study we have utilized the streptomycin mouse model for gut infection to study the role of the Tat system in *Salmonella*-induced colitis. In particular, the treatment with streptomycin can transiently reduce the normal gut microbiota, allowing the elicitation of gut inflammation and gut colonization by *S*. Tm [41, 42]. Using this infection model, we demonstrated that the Tat system is involved in *S*. Tm gut infection by contributing to sustained gut colonization. Furthermore, we showed that Tat substrate-dependent cell division is required for sustained colonization in the inflamed gut, as mutations in AmiA and AmiC cause *S*. Tm cells to form chains by a cell division defect, resulting in their enhanced susceptibility to bile acids. Our findings provide novel insight into *S*. Tm colonization in the inflamed gut and suggest that the Tat system and the control of bacterial cell shape might comprise promising therapeutic targets against gut infection by enteropathogenic bacteria.

## Results

### *S*. Tm Tat is involved in enterocolitis

**Fig 1:**
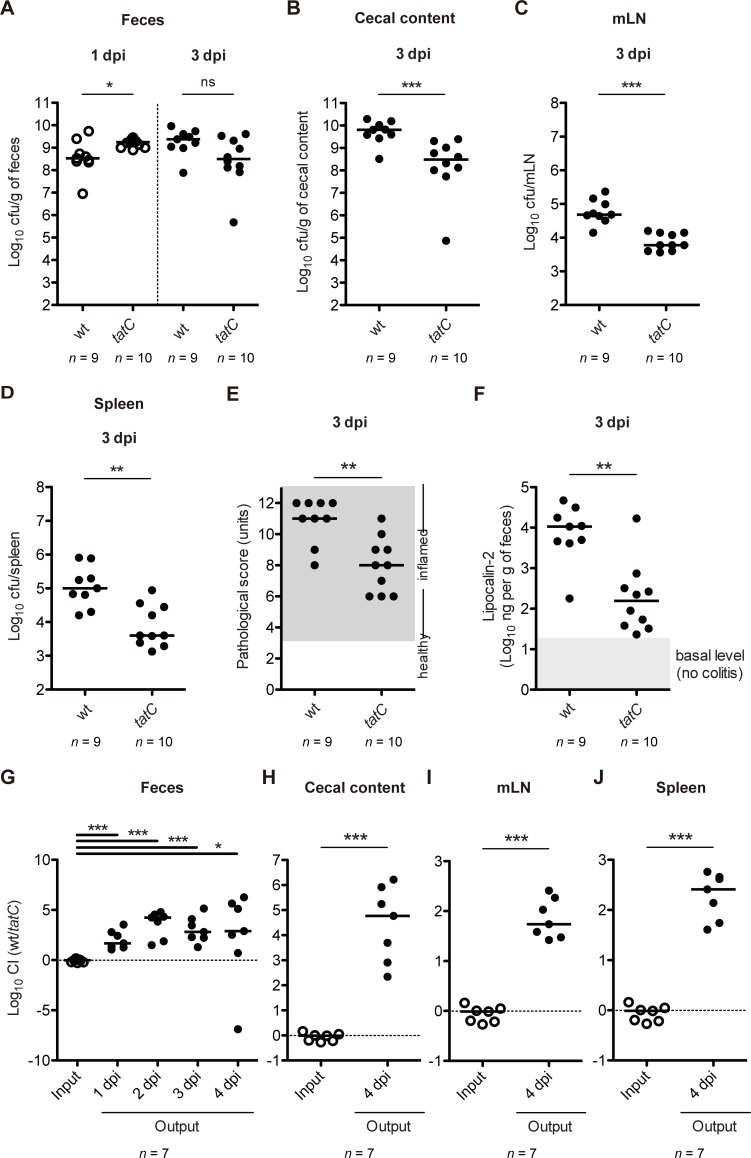
Tat is involved in *S*. Tm-induced colitis. (A-F) Streptomycin-treated C57BL/6 mice (n = 9 or 10 per group) were infected intragastrically for 3 days with 5×10^7^ CFU *S*. Tm wild-type strains SL1344 (wt) or *tatC* mutant (*tatC*). (A) *S*. Tm loads in feces at 1 and 3 days postinfection (dpi). (B) *S*. Tm loads in cecum at 3 dpi. (C) *S*. Tm loads in mesenteric lymph node (mLN) at 3 dpi. (D) *S*. Tm loads in spleen at 3 dpi. (E) Cecal pathological score in H&E-stained cecal tissue section. (F) Fecal lipcalin-2 ELISA. (G-J) Streptomycin-treated C57BL/6 mice (n = 7) were infected intragastrically for 4 days with 1:1 mixture (total 5×10^7^ CFU) of *S*. Tm strains wt and *tatC*. (G) CI of *S*. Tm loads in feces at 1 to 4 dpi. (H) CI of *S*. Tm loads in cecum at 4 dpi. (I) CI of *S*. Tm loads in mLN at 4 dpi. (J) CI of *S*. Tm loads in spleen at 4 dpi. Bar indicates median. ns, not significant; *P < 0.05; **P < 0.01; ***P < 0.001; Mann-Whitney U test.

To reveal the role of Tat in *Salmonella* enterocolitis, we first investigated whether Tat is required for the disease using the streptomycin mouse model. Two groups of streptomycin-pretreated C57BL/6 mice were infected intragastrically with 5 × 10^7^ colony forming units (CFU) of *S*. Tm wild-type strain SL1344 or *tatC* mutant. At day 1 and 3 post infection (p.i.), bacterial loads in feces were enumerated by dilute-plating on selective media. At day 1 p.i., higher loads of *tatC* mutant were detected in feces compared with those of SL1344, whereas both strains at day 3 p.i. displayed high levels of colonization (Fig 1A). The bacterial loads of *tatC* mutant at day 3 p.i. in cecal content, mesenteric lymph node (mLN), and spleen were significantly lower than those in SL1344 (Fig 1B–1D). Histopathological analysis of the cecal mucosa showed that each mouse infected with SL1344 or *tatC* mutant featured gut inflammation (Fig 1E). Notably, however, the mice infected with *tatC* mutant displayed lower levels of gut inflammation in comparison with those infected with SL1344. In addition, the levels of gut inflammation were further verified by determining the amount of fecal lipocalin-2, an inflammatory marker (Fig 1F).

To clarify the role of Tat in gut colonization, we performed a competitive infection (CI) experiment. Streptomycin-pretreated C57BL/6 mice were infected with a 1:1 mixture of SL1344 and *tatC* mutant by gavage (5 × 10^7^ CFU in total). In the feces, the bacterial loads of T321 at day 1–4 p.i. were significantly lower than those of SL1344 (Fig 1G). Similar colonization defects of *tatC* mutant at day 4 p.i. were found in the cecum lumen, mLN, and spleen (Fig 1H–1J). Collectively, these results suggest that *S*. Tm Tat is involved in the *Salmonella* enterocolitis.

### Tat-exported peptidoglycan amidases AmiA and AmiC are involved in *S*. Tm-induced colitis

To further investigate the role of Tat in gut colonization, we used an attenuated *S*. Tm strain harboring a mutation of the *ssaV* gene, which encodes a component of ttss-2, in further experiments. The attenuated strain allowed us to evaluate sustained gut colonization in the streptomycin mouse model [43, 44].

In *Escherichia coli*, *S*. Enteritidis, and *S*. Tm, *tatC* mutant cells have been shown to form long chains resulting from the failure to separate following cell division [38, 40, 45]. Furthermore, it has been reported that *E*. *coli* and *S*. Tm strains lacking certain Tat substrates such as Ami amidases display a cell division defect. [40, 46]. As it is possible that the impaired colonization in the *S*. Tm *tatC* mutant strain might be due to a defect in the export of Tat substrate(s), we focused on two amidases, AmiA and AmiC, as determinants responsible for the Tat-dependent gut colonization. First, we investigated whether the *ssaV tatC* mutant features a cell division defect. In the current experiment, test strain cells harbored a GFP-expressing plasmid (pACYC-gfp), allowing the resulting bacterial strains grown in LB broth to be analyzed by fluorescence microscopy. Approximately 40–60% of the cells of *ssaV* mutant were single or paired, but no chains were observed (S1A and S1B Fig). In contrast, in all cell configurations, the proportion of single cells of *ssaV tatC* mutant was reduced in comparison with that of the *ssaV* mutant, whereas increased proportion of chains was observed (S1A and S1B Fig). Next, we performed the same analysis with *amiA*, *amiC*, or *amiA amiC* mutants. Similar to the *ssaV tatC* mutant, the proportions of single cells of *ssaV amiC* or *ssaV amiA amiC* mutants were reduced, whereas increased chains were observed. (S1A and S1B Fig). In contrast, no difference in *ssaV amiA* mutant was observed (S1A and S1B Fig). Furthermore, the cell division defect of the *ssaV amiA amiC* mutant was restored by introduction of a plasmid encoding the wild-type *amiC*, but not *amiA* gene (S1C Fig). Finally, scanning electron microscopy analysis showed that the *ssaV amiA amiC* mutant strain results in chains, whereas the *ssaV* mutant strain displayed single or paired cells (S1D Fig). These results confirmed that *S*. Tm Tat and its substrates AmiA and AmiC contribute to cell division and that AmiC, but not AmiA, is more likely to be required for the cell division.

**Fig 2:**
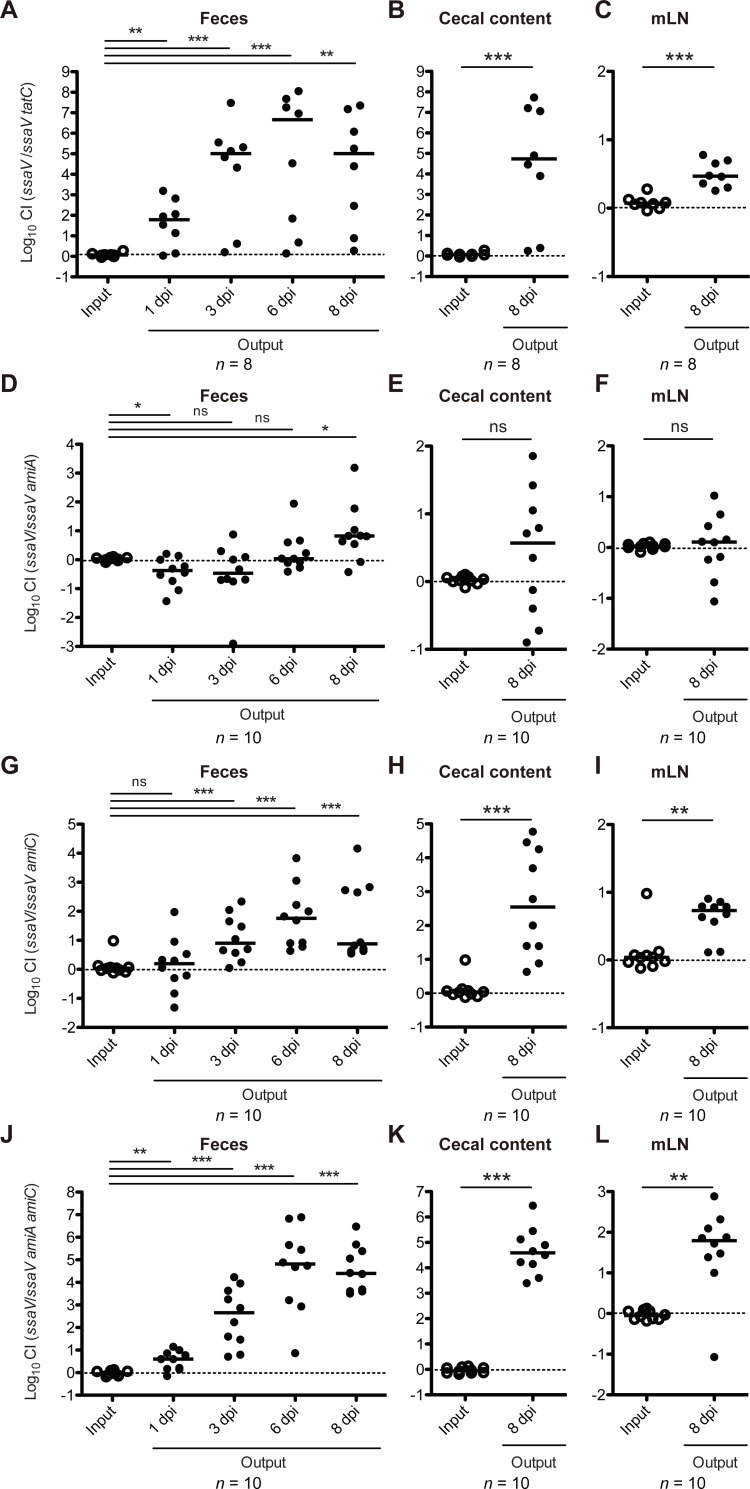
AmiA and AmiC contribute to *S*. Tm gut colonization. Streptomycin-treated C57BL/6 mice (n = 8 or 10) were infected for 8 days with 1:1 mixture (total 5×10^7^ CFU intragastrically) of *S*. Tm *ssaV* and *ssaV tatC* mutants, or *ssaV* and *ssaV amiA* mutants, or *ssaV* and *ssaV amiC* mutants, or *ssaV* and *ssaV amiA amiC* mutants. (A, D, G and J) CI of *S*. Tm loads in feces at 1, 3, 6, and 8 dpi. (B, E, H and K) CI of *S*. Tm loads in cecum at 8 dpi. (C, F, I and L) CI of *S*. Tm loads in mLN at 8 dpi. Bar indicates median. ns, not significant; *P < 0.05; **P < 0.01; ***P < 0.001; Mann-Whitney U test.

Thus, to investigate the role of Tat in sustained gut colonization, we performed a CI experiment using *ssaV* mutant and *ssaV tatC* mutants. The *ssaV tatC* mutant exhibited a substantial colonization defect in the feces at day 1, 3, 6, and 8 p.i. (Fig 2A). The colonization defect was also found in the cecal content and mLN at day 8 p.i. (Fig 2B and 2C). Similarly, we tested *ssaV amiA* or *ssaV amiC* or *ssaV amiA amiC* mutants. In feces, each the *ssaV amiC* and the *ssaV amiA amiC* strains featured a significant colonization defect at day 3, 6, and 8 p.i. (Fig 2G and 2J). Colonization defects in the cecum lumen and mLN by these mutants were also found (Fig 2H, 2I, 2K and 2L). In contrast, in feces, the *ssaV amiA* strain displayed attenuated colonization at day 8 p.i., whereas no colonization defects were found in the cecum lumen or mLN (Fig 2D–2F). In addition, we performed a complementation CI experiment using *ssaV amiC* mutant harboring a plasmid encoding *amiC* gene. Introduction of *amiC* gene rescued the colonization defect of the *ssaV amiC* mutant at day 6 p.i. (S2 Fig). These results suggest that AmiC, and to lesser extent AmiA contribute to gut colonization.

Furthermore, to examine the contribution of AmiA and AmiC to the gut colonization in more detail, we repeated the CI experiments using the complemented *ssaV amiA amiC* strains with *amiA* or *amiC* genes. Introduction with *amiC* gene, but not *amiA*, complemented the colonization defect of the *ssaV amiA amiC* mutant (S3 Fig). The results clearly indicated that AmiC mainly contributes to the gut colonization.

The AmiA- and AmiC-dependent colonization defect was confirmed by comparing the mice infected with the *ssaV* or the *ssaV amiA amiC* mutants (S4A–S4C Fig). Streptomycin-pretreated mice were infected with the *ssaV* or *ssaV amiA amiC* mutant, and gut colonization was monitored for the next 8 days p.i.. At day 1 and 3 p.i., both strains colonized the gut at similar levels (S4A Fig). However, at day 8 p.i., the *ssaV amiA amiC* mutant loads were significantly decreased (S4A Fig). Similar colonization defect of the *ssaV amiA amiC* mutant was found in the cecal lumen (S4B Fig). Histopathological analysis of cecal mucosa showed that mice infected with the *ssaV* mutant exhibited moderate mucosal inflammation whereas the *ssaV amiA amiC* mutant-infected murine cecum displayed only slight inflammation (S4C Fig).

Collectively, these results suggested that the attenuated virulence of the *S*. Tm *tatC* mutant in the enterocolitis model could be explained by an export defect of AmiA and AmiC.

### *S*. Tm *amiA amiC* mutant strain forms chains in the gut

**Fig 3:**
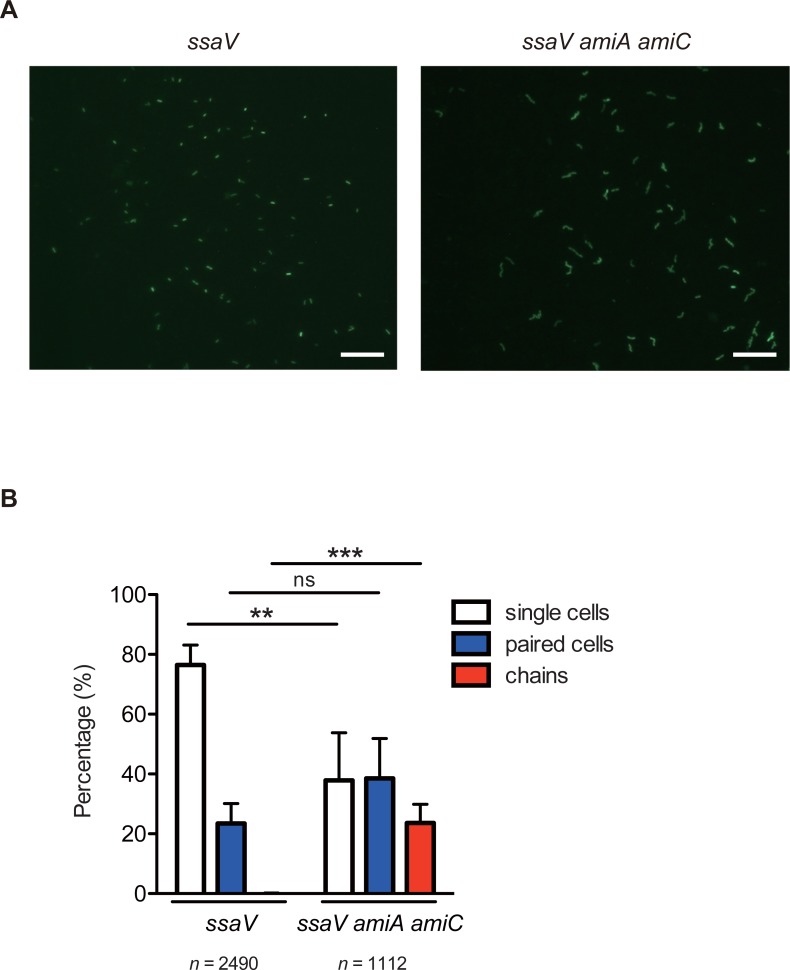
*S*. Tm *amiA amiC* mutant strain displays chains in the inflamed gut. Streptomycin-treated C57BL/6 mice (n = 4 mice per group) were infected by gavage (5×10^7^ CFU) for 3 days with GFP-expressing *S*. Tm *ssaV* or *ssaV amiA amiC* mutants. Fecal pellets were collected, and then resuspended gently with PBS. The resulting *S*. Tm cells were observed by fluorescence microscopy. (A) Representative fluorescence microscopy images of GFP-expressing *S*. Tm in the feces at 3 dpi (400x). Scale bar, 20 μm. (B) Quantitative analyses of the experiments. Data are shown as means ± standard deviations. ns, not significant; **P < 0.01; ***P < 0.001; unpaired Student’s t-test.

To assess whether the *S*. Tm *amiA amiC* mutant strain displays long chains in the gut, streptomycin-pretreated C57BL/6 mice were infected with GFP-expressing *S*. Tm strains *ssaV* or *ssaV amiA amiC* by gavage. For the next 3 days, an obtained fecal pellet was suspended by pipetting gently and analyzed using fluorescence microscopy. Live microscopy analysis revealed that at day 3 p.i., the majority of *ssaV* mutant were present in single or paired cells, whereas the *ssaV amiA amiC* cells exhibited chain formation (Fig 3A). Quantitative analysis of the experiments revealed that approximate 20% of the *S*. Tm cells in the gut were presented in chains at day 3 p.i. (Fig 3B). These results suggest that the *S*. Tm *amiA amiC* mutant forms chain-shaped structures in the gut and may imply that the chain form underlies the attenuated gut colonization observed with this mutant strain.

### Host inflammation is involved in AmiA- and AmiC-dependent competitive fitness

**Fig 4:**
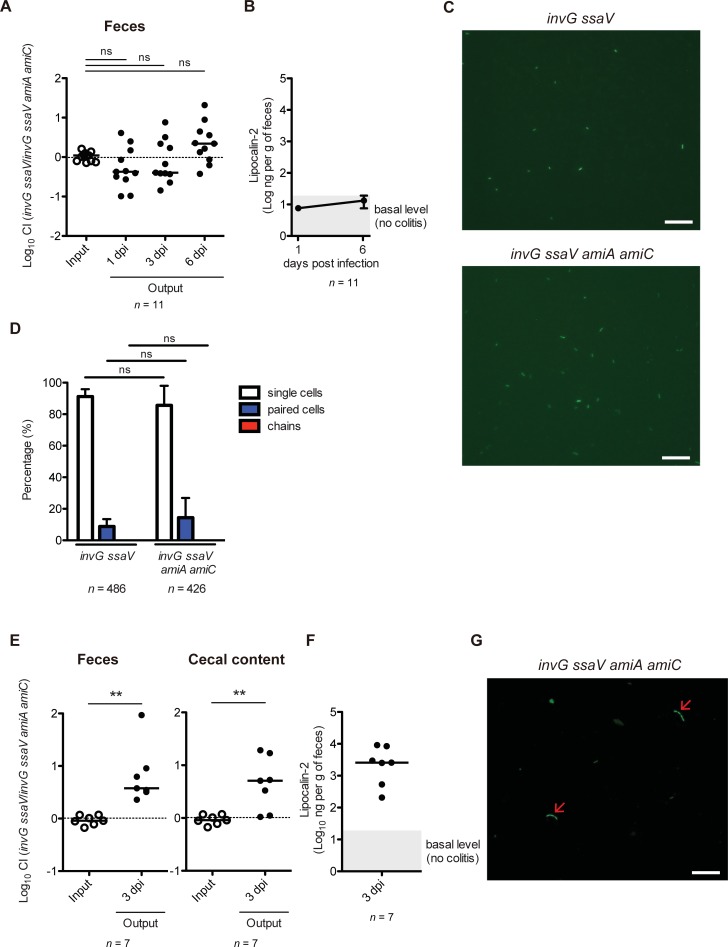
Possible link between host inflammation and AmiA- and AmiC-dependent competitive fitness. (A and B) Streptomycin-treated C57BL/6 mice (n = 11) were infected for 6 days with 1:1 mixture (total 5×10^7^ CFU intragastrically) of *S*. Tm *invG ssaV* and *invG ssaV amiA amiC* strains. (A) CIs of *S*. Tm loads in feces at 1, 3, and 6 dpi were determined. (B) Lipocalin-2 was monitored by ELISA. Data points represents geometric means ± standard deviations. (C) Streptomycin-treated C57BL/6 mice (n = 3 per group) were infected by gavage (5×10^7^ CFU) for 3 days with GFP-expressing *S*. Tm *invG ssaV* or *invG ssaV amiA amiC* strains. Fecal pellets were collected, and then resuspended gently with PBS. The resulting *S*. Tm cells were observed by fluorescence microscopy. Representative fluorescence microscopy images of GFP-expressing *S*. Tm in the feces at 3 dpi (400x). Scale bar, 20 μm. (D) Quantitative analysis of the experiment. Data are shown as means ± standard deviations. ns, not significant; unpaired Student’s t-test. (E and F) Prior to treatment with streptomycin, C57BL/6 mice (n = 7) were exposed to 3.5% DSS *ad libitum* for 7 days. Subsequently, mice were treated with streptomycin, followed by mixed infection with *invG ssaV* and *invG ssaV amiA amiC* mutants via oral route. (E) CIs of *S*. Tm loads in feces or the cecum at 3 dpi were determined. (F) DSS-induced inflammation was verified by Lipocalin-2 ELISA. (G) Prior to treatment with streptomycin, C57BL/6 mice (n = 3) were exposed to 3.5% DSS *ad libitum* for 4 days, and treated with streptomycin, followed by infection by gavage (5×10^7^ CFU) with GFP-expressing *S*. Tm *invG ssaV amiA amiC* mutant. Fecal pellets were collected, and then resuspended gently with PBS. The resulting cells were observed by fluorescence microscopy. Representative fluorescence microscopy images of GFP-expressing *S*. Tm in the feces at 3 dpi (400x). Arrow indicates chain form of *S*. Tm cells. Scale bar, 20 μm. For (A), (E) and (F), bar indicates median. ns, not significant; **P < 0.01; Mann-Whitney U test.

*S*. Tm exploits host inflammatory responses to colonize in the gut [34], which suggests that gut inflammation is required for the competitive fitness advantage of *S*. Tm. This prompted us to hypothesize that gut inflammation is also involved in the AmiA- and AmiC-dependent competitive fitness. To this end, we performed a CI experiment using the *S*. Tm *invG ssaV* avirulent strain, which is incapable of inducing the gut inflammation [47]. We first confirmed that a mutation of the *invG* gene had no effect on the cell division *in vitr*o (S5 Fig). Streptomycin-pretreated C57BL/6 mice were co-infected with *invG ssaV* and *invG ssaV amiA amiC* strains at a 1:1 mixture by gavage (5 × 10^7^ CFU in total), and *S*. Tm loads in feces were monitored at day 1, 3, and 6 p.i.. Both strains displayed similar colonization levels at all monitored days (Fig 4A). The absence of gut inflammation in the mice was verified by measuring fecal Lcn-2 levels (Fig 4B). Next, we examined whether *S*. Tm *amiA amiC* mutant cells could form chains via the cell division defect in the normal gut using the GFP-expressing *S*. Tm. Live microscopy and quantitative analyses revealed that all of the *invG ssaV* and *invG ssaV amiA amiC* cells were present as single or paired cells in the gut, whereas no chains were observed (Fig 4C and 4D). The results demonstrated that the cell division defect of the *amiA amiC* mutant strain does not occur in the normal gut.

Next, to analyze the relevance of gut inflammation in supporting *S*. Tm colonization, we further investigated whether dextran sulfate sodium (DSS) could confer a colonization defect on the *invG ssaV amiA amiC* strain in the CI experiment. C57BL/6 mice with DSS-induced colitis were subjected to the same CI experiment as shown in Fig 4A. Effect of the DSS treatment was verified by measuring body weight of the mice, suggesting that DSS treatment for 7 days appears to induce colitis (S6 Fig). In addition, the DSS-treated mice were administered orally with streptomycin prior to infection in order to reduce competitive microbiota. Colonization levels at day 3 p.i. of the *invG ssaV amiA amiC* strain in feces and cecal content were significantly reduced (Fig 4E). Gut inflammation in the mice was confirmed by elevated fecal Lcn-2 levels (Fig 4F). Finally, we investigated whether the *invG ssaV amiA amiC* strain cells could form chains in the DSS-induced inflamed gut. As shown in Fig 4G, the *invG ssaV amiA amiC* strain formed chains when used to infect the DSS-treated mouse. The results suggested that the *invG ssaV amiA amiC* mutant was incapable of fully dividing cells in certain conditions such as in the inflamed gut. Collectively, these results indicated that host inflammatory responses may be involved in the AmiA- and AmiC-dependent competitive fitness.

### The colonization defect of the *S*. Tm *amiA amiC* mutant strain is not associated with impaired motility

**Fig 5:**
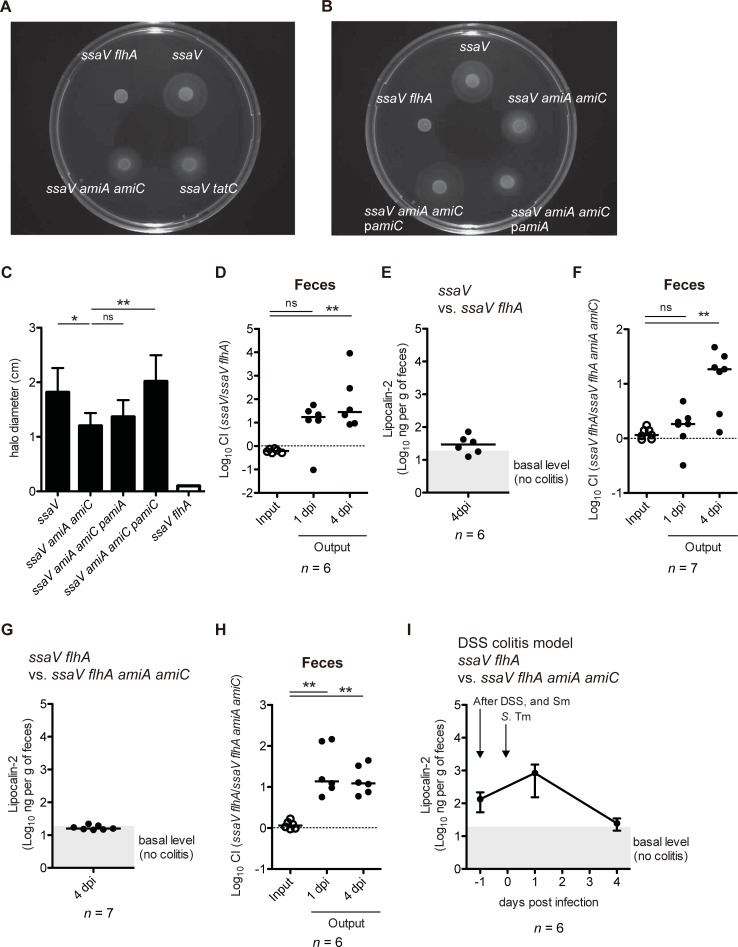
Non-motile *S*. Tm strain display the AmiA- and AmiC-dependent colonization defect. (A and B) *S*. Tm strains were plated on LB with 0.3% agar (swimming), and incubated at 37°C for 5 hours. (C) The halo was measured. The assay was performed six times independently. Data are shown as means ± standard deviations. ns, not significant; *P < 0.05; **P < 0.01; unpaired Student’s t-test. (D-G) Streptomycin-treated C57BL/6 mice (n = 6 or 7) were infected for 4 days with 1:1 mixture (total 5×10^7^ CFU intragastrically) of *S*. Tm *ssaV* and *ssaV flhA* mutants or *ssaV flhA* and *ssaV flhA amiA amiC* mutants. (D and F) CIs of *S*. Tm loads in feces at 1 and 4 dpi were determined. (E and G) Fecal lipocalin-2 ELISA. (H and I) Prior to treatment with streptomycin, C57BL/6 mice (n = 6) were exposed to 3.5% DSS *ad libitum* for 4 days. Subsequently, mice were treated with streptomycin, followed by mixed infection with *ssaV flhA* and *ssaV flhA amiA amiC* mutants via oral route. (H) CIs of *S*. Tm loads in feces at 1 and 4 dpi were determined. (I) Lipocalin-2 ELISA, showing that DSS treatment elicits inflammation. Sm, streptomycin. Bar indicates median. ns, not significant; **P < 0.01; ***P < 0.001; Mann-Whitney U test.

To decipher the molecular mechanism underlying the AmiA- and AmiC-dependent competitive fitness advantage, we noted phenotypes that were modified following mutation of the *amiA* and *amiC* genes. Previously, motility has been shown to be attenuated by deletions of the *tatC* gene or both *amiA* and *amiC* genes in *S*. Tm strain 14028 [39, 40]. Furthermore, motility was the first phenotype identified that allowed *S*. Tm to capitalize on mucosal inflammation, resulting in sustained gut colonization [27]. Thus, we investigated role of motility in the AmiA- and AmiC-dependent competitive fitness. First, we examined the ability of *ssaV*, *ssaV tatC*, or *ssaV amiA amiC* mutants to move on semi-agar (0.3% agar) medium. The *ssaV* mutant were motile in comparison with *ssaV flhA* mutant as the negative control, which lacked an inner membrane protein of flagella (Fig 5A). In contrast, the motility of *ssaV tatC* and *ssaV amiA amiC* mutant cells was significantly attenuated, albeit not diminished (Fig 5A). This impaired motility in the *ssaV amiA amiC* mutant was complemented by introduction of a plasmid encoding the *amiC* gene, but not *amiA* (Fig 5B and 5C). In addition, we examined swarming motility using swarming agar (0.5% agar) medium. The *ssaV* mutant cells were motile on the swarming agar, whereas *ssaV cheY* mutant cells swarmed poorly (S7A Fig). In contrast, *ssaV tatC* and *ssaV amiA amiC* strain cells were motile on the swarming agar, and the plasmid-based expression of AmiA or AmiC displayed no effect on the swarming (S7B Fig). These results are in line with the previous reports [39, 40].

To clarify the causal link between the impaired motility of the *ssaV amiA amiC* strain and the colonization defect, we first confirmed whether impaired motility confers colonization defect using the *ssaV flhA* mutant. In line with previous studies [27, 48], in a CI experiment, the *ssaV flhA* mutant displayed colonization defect (Fig 5D). ELISA with fecal pellet showed that the mice in this CI experiment feature slight inflammation (Fig 5E). Thus, we next performed a CI assay using two *S*. Tm strains *ssaV flhA* and *ssaV flhA amiA amiC*. At day 1 p.i., both strains displayed similar colonization levels (Fig 5F). However, at day 4 p.i., the *ssaV flhA amiA amiC* strain showed impairment in competitive colonization (Fig 5F). Accordingly, measurement of fecal Lcn-2 levels suggested that the infected mice did not develop gut inflammation (Fig 5G). Furthermore, to reveal the involvement of directional movement, termed chemotaxis, in the gut colonization by *S*. Tm, we performed a similar CI experiment by *ssaV cheY* and *ssaV cheY amiA amiC* mutants. At day 1 and 4 p.i., colonization levels of the *ssaV cheY amiA amiC* mutant were significantly reduced (S7C Fig). Fecal Lcn-2 measurement of the infected mice suggested that the infected mice demonstrated only minimal inflammation (S7D Fig). These results showed that the non-motile or non-chemotactic *S*. Tm strains still exhibit the AmiA- and AmiC-related colonization defect in the noninflamed gut, suggesting that impaired motility alone is not sufficient to explain the attenuated colonization of the *S*. Tm *amiA amiC* mutant strain.

Because mice in the experiments of Fig 5F and 5G exhibited little gut inflammation, we could not exclude the possibility that bacterial motility is involved in the AmiA- and AmiC-dependent competitive fitness in the inflamed gut. Therefore, to overcome this limitation, we applied the DSS colitis model in the same CI experiment as shown in Fig 5F and 5G. In DSS-induced colitis mice, the *ssaV flhA amiA amiC* mutant was impaired in gut colonization (Fig 5H). Lcn-2 ELISA indicated that DSS treatment elicits inflammation, and that the mice in this CI experiment have gut inflammation during the *S*. Tm infection (Fig 5I). The results indicate that flagella-based motility may be not involved in the AmiA- and AmiC-dependent competitive fitness in the inflamed gut.

### The colonization defect of the *S*. Tm *amiA amiC* mutant strain is conferred by attenuated resistance to bile acid

Antimicrobial molecules such as antimicrobial peptides are believed to play a crucial role in the competitive bacterial fitness of the intestinal tract [49]. Recently, we have shown that *S*. Tm gut colonization requires a robust outer membrane to confer resistance to α-helical antimicrobial peptide [44]. Thus, to clarify the involvement of resistance to antimicrobial peptide in the AmiA- and AmiC-dependent competitive fitness, we next investigated the *S*. Tm *amiA amiC* mutant strain for sensitivity to magainin 2, an α-helical antimicrobial peptide, by determining the minimal inhibitory concentrations (MICs) of magainin 2 towards *S*. Tm strains (Table 1). *S*. Tm *ssaV phoP* mutant featuring an attenuated outer membrane barrier was used as a control. As expected, the MICs of the *ssaV phoP* mutant were reduced in comparison to the SL1344 wild-type strain and *ssaV* mutant. In contrast, the MICs towards the *ssaV tatC* and *ssaV amiA amiC* strains were identical for the *ssaV* mutant. Collectively, these data suggest that the *amiA amiC* mutant strain is resistant to α-helical antimicrobial peptide.

**Table 1:** MICs of antimicrobials towards *S*. Tm.

Strain	Genotype	MICs of magainin-2	MICs of deoxycholate
SL1344	wild-type	512 μg/ml	6%
T145	*ssaV*	512 μg/ml	6%
T146	*ssaV phoP*	64 μg/ml	0.4%
T434	*ssaV tatC*	512 μg/ml	1.2%
T303	*ssaV amiA amiC*	512 μg/ml	0.8%
T410	*ssaV amiA amiC* p*amiA*	512 μg/ml	0.8%
T411	*ssaV amiA amiC* p*amiC*	512 μg/ml	2%

Each experiment was repeated three times independently.

Earlier studies have demonstrated that *tatC* or *amiA* and *amiC* mutants in *E*. *coli* or *S*. Tm are hypersensitive to detergents such as sodium dodecyl sulfate and bile acids [39, 40, 45, 50]. Furthermore, resistance to bile acids has been shown to confer a competitive fitness advantage on *S*. Tm [51]. Thus, we next tested for sensitivity to deoxycholate, a component of bile acid detergents. We determined the MICs of deoxycholate towards *S*. Tm strains (Table 1). Compared to SL1344 and *ssaV* mutant, the MICs of deoxycholate for *ssaV phoP* or *ssaV tatC* or *ssaV amiA amiC* mutants were reduced. In contrast, the MICs of complemented *S*. Tm strains with *amiC*, but not *amiA*, were restored partially. Collectively, these data demonstrated that *S*. Tm strains harboring mutations of *tatC* or *amiC* genes are sensitive to bile acid such as deoxycholate. Furthermore, it is notable that AmiC, but not AmiA, contributes to the resistance of *S*. Tm to bile acids.

**Fig 6:**
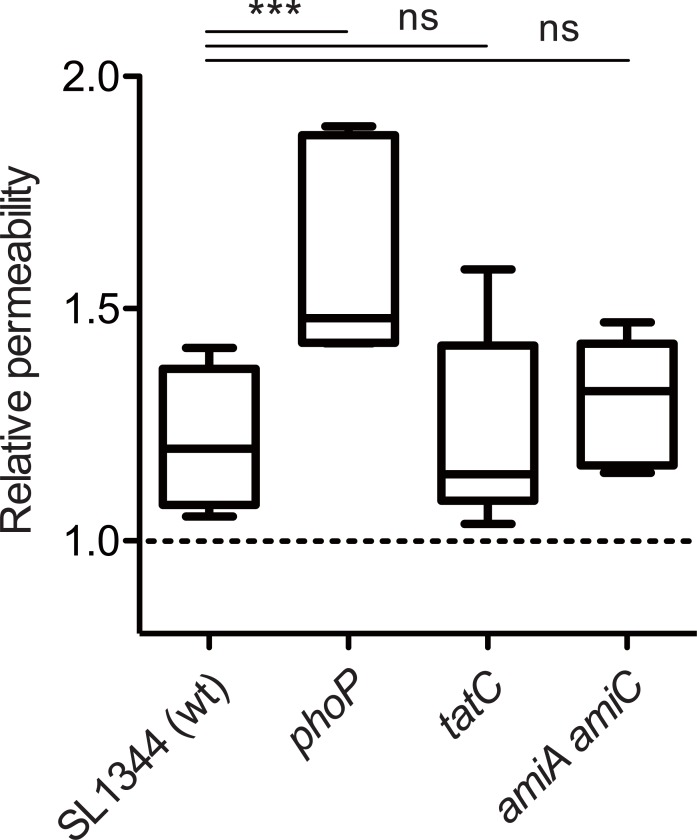
AmiA and AmiC do not contribute to construction of robust outer membrane. Outer membrane permeability was measured using the EtBr influx assay, evaluated by comparing the sample without *S*. Tm, meaning that more than 1 indicates outer membrane permeability was increased [44, 53]. Five independent experiments were performed. Box & whiskers plot: the whiskers denote minimum and maximum, and the black bars indicate medians. ns, not significant (*P* ≥ 0.05); ***P < 0.001; Mann-Whitney U test.

Since resistance of *S*. Tm to bile acids generally depends upon the robust outer membrane [52], we next assessed bacterial outer membrane barrier by using the ethidium bromide (EtBr) influx assay [44]. If the outer membrane barrier is attenuated, EtBr can pass through the outer membrane easily, and subsequently reach the cytosol by traversing the cytoplasmic membrane. The EtBr in the bacterial cytosol leads to an increase in fluorescence signal by binding to intracellular nucleic acids. Compared to SL1344, *S*. Tm *phoP* mutant cells displayed the increased fluorescence intensity (Fig 6). In contrast, the intensity of *tatC* or *amiA amiC* mutant cells was identical for that of SL1344. These results suggest that AmiA and AmiC do not contribute to the robustness of the outer membrane, and that the outer membrane barrier does not involve the impaired resistance of the *amiA amiC* mutant strain towards to bile acids.

Next, we investigated whether attenuated resistance to bile acid confers impaired gut colonization on the *S*. Tm *amiA amiC* mutant strain by using rodent chow containing cholestimide resin, which enhances the excretion of bile acids in feces via absorption in the intestinal tract, limiting the intestinal circulation of bile acids [54–56]. Total bile acids concentrations in feces of mice fed chow containing colestimide increased significantly compared to those of mice fed control chow (S8A and S8B Fig). The results indicated that feeding with chow containing colestimide resin promotes the excretion of luminal bile acids in feces through the adsorption of the colestimide resin to bile acids, leading to a reduction in the concentrations of free luminal bile acids, which can interact with luminal substances including bacteria. Thus, we confirmed an inhibitory effect on luminal bile acids by feeding with colestimide resin.

**Fig 7:**
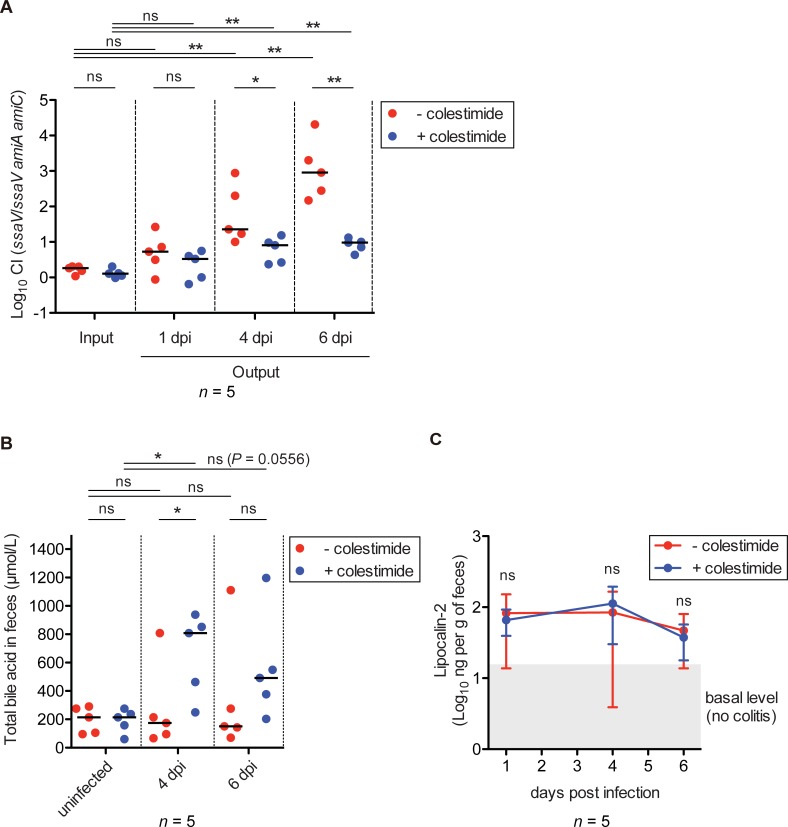
Feeding with the bile sequestrant cholestimide resin reduces the AmiA- and AmiC-dependent competitive fitness advantage. C57BL/6 mice (n = 5 per group) fed chow containing cholestimide resin or control chow were treated with streptomycin and infected for 6 days with 1:1 mixture (total 5×10^7^ CFU intragastrically) of *S*. Tm *ssaV* and *ssaV amiA amiC* strains. (A) CIs of *S*. Tm loads in feces at 1, 4, and 6 dpi were determined. (B) Concentrations of total bile acids in feces. (C) Fecal lipocalin-2 ELISA. For (A) and (B), bar indicates median. For (C), data points represent means ± standard deviations. ns, not significant; *P < 0.05; **P < 0.01; Mann-Whitney U test.

C57BL/6 mice were then fed chow containing cholestimide resin or control chow, and subjected to the streptomycin mouse model experiment with mixed infection of *S*. Tm *ssaV* and *ssaV amiA amiC* mutants. In mice fed normal chow, competitive colonization advantages of *ssaV* mutant increased gradually compared to input CI (Fig 7A). In contrast, the advantages were slightly increased compared to input CI in mice fed chow containing cholestimide resin, and were significantly reduced compared to mice fed normal chow at day 4 and 6 p.i. (Fig 7A). *S*. Tm-infected mice fed control chow displayed no increase in the concentration of total bile acids in feces, whereas in the mice fed chow containing cholestimide, total bile acids in feces increased significantly at day 4 p.i. (Fig 7B). Furthermore, feeding with chow containing cholestimide resin tended to increase the concentrations of total bile acids of mice infected with *S*. Tm at day 6 p.i. (Fig 7B). The results indicated that luminal colestimide resin binds to bile acids and thereby forms an insoluble complex, resulting in a decrease in the free luminal bile acids that can interact with *S*. Tm and an increase in total bile acids in feces. Finally, measurement of fecal lipocalin-2 levels suggested that both mouse groups exhibited gut inflammation and that no difference in inflammation levels existed between the two groups (Fig 7C). Collectively, these results lend support to the hypothesis that the gut colonization defect of *S*. Tm *tatC* or *amiA amiC* mutant strains is attributable to attenuated resistance to bile acids.

### High osmolarity and antimicrobial peptides impose AmiA- and AmiC-dependent cell division on *S*. Tm

**Fig 8:**
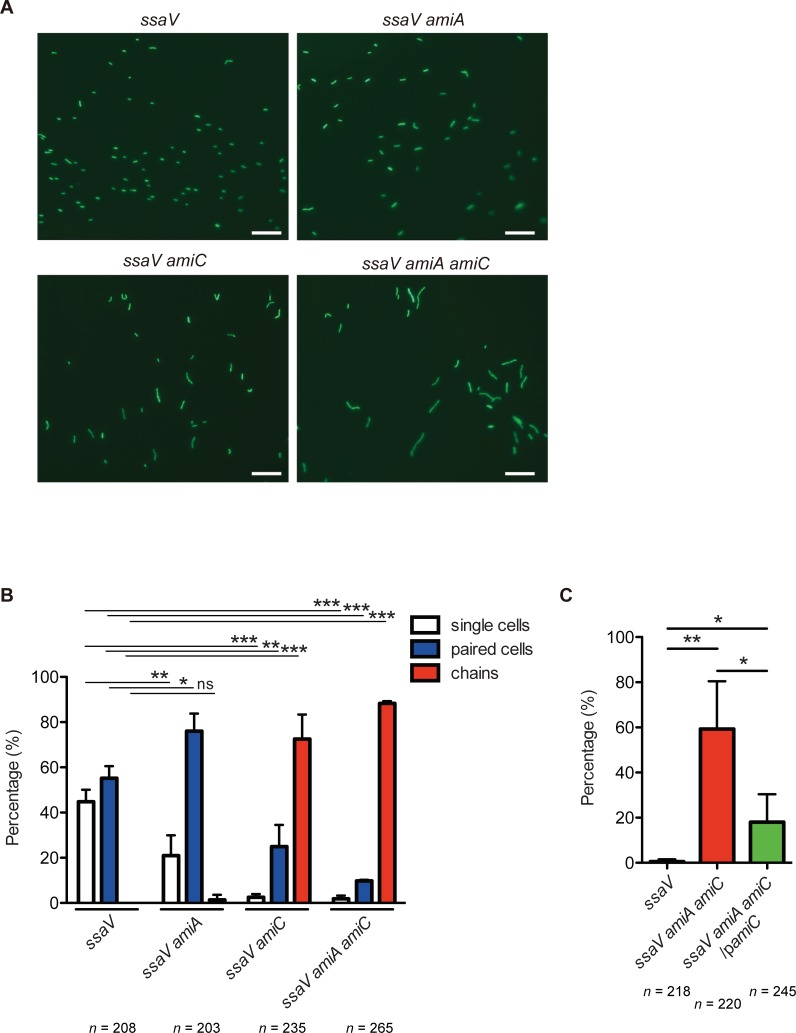
High osmolarity and antimicrobial peptide impose AmiA- and AmiC-dependent cell division. GFP-expressing *S*. Tm *ssaV*, *ssaV amiA*, *ssaV amiC*, or *ssaV amiA amiC* strains were grown in LB with 0.5 M NaCl. *S*. Tm cells were placed on a 1.5% agarose pad, sealed under a glass coverslip, and observed by fluorescence microscopy. (A) Representative fluorescence microscopy images of GFP-expressing *S*. Tm strains grown in LB with 0.5 M NaCl (400x). Scale bar, 20 μm. (B) Quantitative analyses of the experiment. Bars represent mean ± SD from three independent experiments. ns, not significant; *P < 0.05; **P < 0.01; ***P < 0.001; unpaired Student’s t-test. (C) *S*. Tm *ssaV*, *ssaV amiA amiC*, or *ssaV amiA amiC* harboring p*amiC* strains grown in LB supplemented with 1 μg/m polymyxin B were observed by light microscopy. Proportion of chains. Bars represent mean ± SD from four experiments. *P < 0.05; **P < 0.01; unpaired Student’s t-test.

The above data suggested the possibility that certain environmental cues might impose AmiA- and AmiC-dependent cell division in the inflamed gut. To address this issue, we examined the expression pattern of *amiA* and *amiC* genes. As *S*. Tm *amiA* and *amiC* expression is positively regulated by the CpxRA two-component system [57], the bacterial strains were grown under CpxRA-inducible conditions; i.e., high osmolarity (here, 0.5 M NaCl) [58] or the presence of antimicrobial peptide (here, 1 μg/ml polymyxin B) [47, 59], and subjected to microscopy analysis. When grown in LB broth containing 0.5 M NaCl, in all cell configurations, the proportion of chains of GFP-expressing *ssaV amiC* and GFP-expressing *ssaV amiA amiC* strains were dramatically increased (Fig 8A and 8B). Similar results were obtained upon growth in LB broth containing polymyxin B (S9 Fig). This was confirmed by a complementary experiment, showing that the introduction of a plasmid expressing AmiC *in trans* partially restored cell division (Fig 8C). These results indicated that high osmolarity and the presence of antimicrobial peptides, which comprise a similar condition to that of the gut lumen, impose AmiA- and AmiC-dependent cell division.

### The chain form confers impaired resistance to deoxycholate on *S*. Tm

The previous results suggest that the chain form of the *S*. Tm *amiA amiC* mutant strain may confer impaired resistance to bile acids. To clarify this possibility, we examined a causal link between the chain form and bile acid resistance by comparing *S*. Tm cells grown in LB medium with those grown in LB plus 0.5 M NaCl.

**Fig 9:**
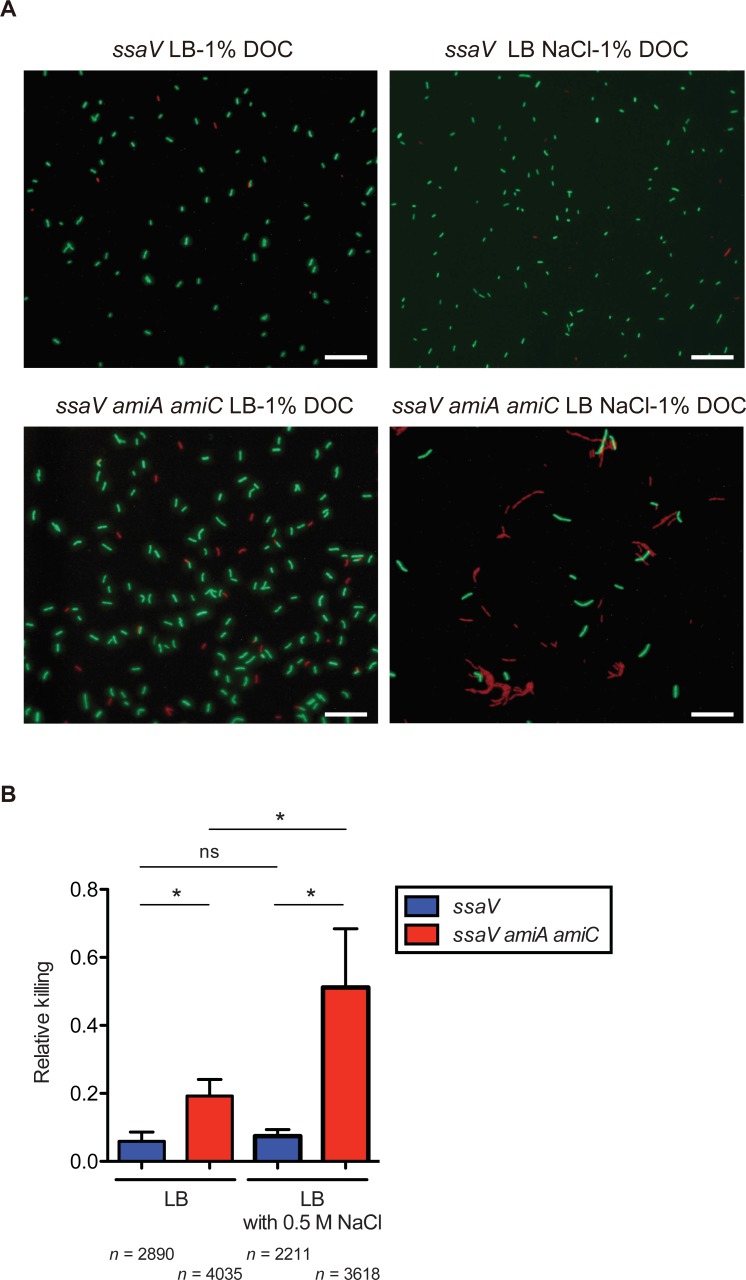
Chain form confers attenuated resistance to deoxycholate. GFP-expressing *S*. Tm *ssaV* or *ssaV amiA amiC* mutants were grown in LB or LB plus 0.5 M NaCl, and subsequently were mixed with 1% deoxycholate (DOC), followed by incubation and treatment with PI. The resulting *S*. Tm cells were placed on a 1.5% agarose pad, sealed under a glass coverslip, and observed by fluorescence microscopy. (A) Representative fluorescence microscopy images. Scale bar, 20 μm. (B) Microscopy quantification of PI-stained cells. Three independent experiments were performed. ns, not significant; *P < 0.05; unpaired Student’s t-test.

GFP-expressing *S*. Tm *ssaV* or *ssaV amiA amiC* strains grown in LB medium or LB plus 0.5 M NaCl were incubated with 1% deoxycholate, with the killing effect subsequently evaluated by using the membrane integrity indicator dye, propidium iodide (PI). If the bacterial membrane was damaged by deoxycholate, *S*. Tm would not be able to express GFP, resulting in PI-stained cells. Consistent with the previous results of MICs (Table 1), GFP-expressing *ssaV* cells grown in both LB medium and LB plus 0.5 M NaCl were resistant to 1% deoxycholate (Fig 9A and 9B). In contrast, approximately 20% of the *ssaV amiA amiC* mutant cells grown in LB medium were killed by 1% deoxycholate. Furthermore, the proportion of deoxycholate-killed (PI-stained) cells in the *ssaV amiA amiC* mutant cells grown in LB plus 0.5 M NaCl was significantly increased (Fig 9A and 9B). These results suggest that the chain form of the *S*. Tm *amiA amiC* mutant strain is more susceptible to deoxycholate-mediated bactericidal effect than the single or paired cells, and that the chain form therefore likely confers attenuated resistance to bile acids on luminal *S*. Tm.

## Discussion

The Tat system is widely conserved in many bacterial pathogens and plays crucial roles in virulence [25]. Therefore, it is expected that the Tat system may represent a therapeutic target for bacterial pathogen infection. Earlier studies revealed that the *S*. Tm *tatC* mutant exhibits attenuated virulence [39], consequent to an export defect of certain Tat substrates including AmiA and AmiC [40]. However, the lack of intestinal inflammation in the previously utilized *Salmonella* typhoid fever mouse model precluded determination of whether the Tat system is involved in *Salmonella*-induced enterocolitis. Thus, to clarify the role of the Tat system in *Salmonella* enterocolitis, in this study we utilized the streptomycin mouse model [28, 41, 60] in which infected *S*. Tm induces severe intestinal inflammation and colonizes the gut [28, 60]. Our results showed that the *S*. Tm *tatC* mutant can elicit intestinal inflammation, albeit at a lower level as compared to that of the wild-type strain SL1344. This appears to be correlated with the colonization levels in the cecum, which exhibited attenuated colonization of the *S*. Tm *tatC* mutant. As deletion of the *tatC* gene causes reduced expression of the *hilA* gene, which encodes a central regulator for ttss-1 [40], the attenuated inflammation might be due to reduced expression of ttss-1. Moreover, we also demonstrate here that gut colonization of the *S*. Tm *tatC* mutant is attenuated in a mixed infection experiment. Based on our data, we concluded that the Tat system in *S*. Tm is not essential for colitis but is involved in the induction of gut inflammation and colonization in the early infectious course.

To date, numerous substrates (approximately 30 proteins) exported by the *S*. Tm Tat machinery have been identified and predicted according to the presence of Tat signal peptides [4]. Conversely, the *S*. Tm *tatC* mutant cannot export any substrates, thereby rendering this mutation likely to confer attenuated virulence. Earlier research showed that the attenuated virulence of the *S*. Tm *tatC* mutant strain in the *Salmonella* typhoid fever model could mainly be attributable to an export defect of AmiA, AmiC and SufI [40]. Here, we demonstrate that in the *Salmonella* enterocolitis model, the attenuated gut colonization of the *S*. Tm *tatC* mutant is attributable to an export defect of AmiA and AmiC. Moreover, single mutation of the *amiA* gene contributes little to the virulence, whereas combined mutations of *amiA* and *amiC* genes lead to significant attenuation of the gut colonization compared to that from single mutation of the *amiC* gene, suggesting that the roles of AmiA and AmiC in the virulence are redundant. In contrast, our results with complemented strains suggest that only AmiC is involved in certain phenotypes; i.e., the cell division defect, attenuated motility, and high sensitivity to deoxycholate observed in the *S*. Tm *amiA amiC* mutant strain. Conceivably, a difference in localization of these amidases might cause such distinct roles. Although both AmiA and AmiC act in the periplasmic space, AmiC is specifically localized to the septal ring during cytokinesis [61, 62]. This suggests that the attenuated virulence of the *S*. Tm *tatC* mutant may be mainly attributable to the loss of properly localized AmiC.

Here, we demonstrate that the bactericidal action of bile acids is responsible for impaired luminal growth of the *S*. Tm *amiA amiC* mutant strain in the mouse colitis model. Bile acids act as a detergent that solubilizes fats in intestinal tract. In addition, another important role of bile acids *in vivo* is to affect the luminal bacterial community via potent antimicrobial properties that are mainly explained by membrane damage [63]. This apparently contributes to host mucosal defense against enteropathogens by bacterial killing. In contrast, certain enteropathogenic bacteria are known to be highly resistant to bile acids; for example, *Salmonella* spp., *E*. *coli*, *Campylobacter jejuni*, *Listeria monocytogenes*, and *Clostridium perfringens* [63]. Therefore, it is tempting to suspect that the bile acid tolerance of enteropathogenic bacteria including *S*. Tm represents a critical factor that determines the competitive fitness advantage in the gut. Consistent with this, bile acid tolerance conferred by very long O-antigen contributes to the luminal fitness of *S*. Tm [51]. These observations therefore suggest that agents that cause the attenuation of bile resistance in enteropathogenic bacteria might constitute new promising antimicrobials.

Our data show that the chain form of *S*. Tm cells confers attenuated resistance to bile acids, leading to impaired colonization in the inflamed gut. In the case of Gram-negative bacteria, robustness of the outer membrane plays a critical role in resistance to bile acids; thus, changes in membrane architecture and composition often bring about a bile resistance defect. For example, bacterial freezing, which causes structural damage of the outer membrane, leads to an increase in the susceptibility of *E*. *coli* to bile salts [64]. Furthermore, in *Lactobacillus acidophilus*, changes in fatty acids composition are related to the susceptibility to bile acids through the enhancement of lipid membrane stability [65]. In line with previous reports, our data from this study show that *S*. Tm *phoP* mutants containing an attenuated outer membrane barrier are highly susceptible to bile acids. In contrast, our data also show that even though the outer membrane barrier of the *S*. Tm *amiA amiC* mutant strain is as robust as that of the wild-type strain, this mutant strain displays impaired resistance to bile acids. These findings indicate that the robustness of the outer membrane might be not related to the bile acids resistance of *S*. Tm *amiA amiC* strain. Therefore, at present, it remains unclear why the chain form of the *S*. Tm *amiA amiC* mutant strain lacks the ability to resist killing by bile acids. Deciphering the mechanism underlying attenuated resistance to bile acids in the chain form of *S*. Tm thus deserves further investigation.

Recently, vaccine-induced IgA has been shown to contribute to *S*. Tm elimination from the gut lumen by enchaining the pathogen [66]. This high-affinity IgA forms large monoclonal clumps by coating and cross-linking the *S*. Tm cells in the gut, resulting in attenuated tissue invasion and accelerated elimination of *S*. Tm. This accelerated elimination is attributable to an increase in the rate of clonal extinction. Based on this novel role of IgA [66], we suspect that the *S*. Tm *tatC* or *amiA amiC* mutant may also be eliminated from the gut lumen by the same mechanism.

Notably, the chain form of the *S*. Tm *amiA amiC* mutant strain is induced in the inflamed gut but not in normal gut, indicating that AmiA- and AmiC-dependent cell division is required in the inflamed gut. The question therefore arises regarding which environmental signal(s) in the inflamed gut impose the cell division on *S*. Tm. Our results from the *in vitro* experiments presented here indicate the possibility that high osmolarity and the presence of antimicrobial peptide induce AmiA- and AmiC-dependent cell division in the gut, as the gut luminal osmolarity is known to be quite high (0.3 M NaCl or higher) [67] and antimicrobial peptides are constitutively present in the gut lumen [49]. Considering that during very high osmotic stress (1.2 M NaCl or higher), *S*. Tm undergoes filamentous growth *in vitro* accompanied with changes of outer membrane integrity [68], osmotic stress may generally induce a cell division defect in this bacterium. Furthermore, the CpxRA envelope stress response, which is activated by high osmolarity and antimicrobial peptides [47, 58, 59, 69], has been shown in turn to activate transcription of the *amiA* and *amiC* genes in response to periplasmic stress, which occurs in the inflamed gut [47, 57], and contribute to *S*. Tm gut colonization during *Salmonella*-induced colitis [47]. These findings suggest that environmental stresses such as high osmolarity and antimicrobial peptide in the inflamed gut may elicit envelope perturbations of *S*. Tm, leading to an increase in *amiA* and *amiC* expression in a CpxRA-dependent manner. Although the induced peptidoglycan amidases likely allow *S*. Tm to cope with the environmental stress, the envelope perturbation is likely to induce a cell division defect. Deciphering the mechanism that specifically imposes AmiA- and AmiC-dependent cell division will be an important topic for future work.

In conclusion, we demonstrate that the Tat system and the Tat-exported peptidoglycan amidases, AmiA and AmiC, constitute virulence factors in *Salmonella*-induced enterocolitis. In turn, they also represent promising therapeutic targets against *Salmonella* gut infection. Moreover, controlling bacterial cell shape by inhibiting certain types of cell division might constitute a new therapeutic intervention strategy against infection with bacterial pathogens.

## Methods

### Ethics statement

All animal experiments were approved by the Kitasato University Institutional Animal Care and Use Committee (Permit Number: A13-6, J96-1, J13-1, 17–52, 17–54 and 17–55).

### Bacterial strains and plasmids

Bacterial strains and plasmids used in this study are listed in Table 2. *S*. Tm strain SL1344 is wild-type and a mouse virulent. *S*. Tm strains harboring chromosomal in-frame deletions were created using lambda/red homologous recombination system [70]. Primers used for construction of the mutant strains are listed in S1 Table.

**Table 2:** Strains and plasmid used in this study.

**Strain**	**Genotype**	**Reference**
***Salmonella enterica* serovar Typhimurium**		
SL1344	Wild-type *S*. Typhimurium, *hisG*	[71]
T321	SL1344 Δ*tatC*::*kan*	This study
T145	SL1344 Δ*ssaV*::*cat*	[44]
T434	SL1344 Δ*ssaV* Δ*tatC*::*kan*	This study
TM131	SL1344 Δ*ssaV*	[72]
TM796	SL1344 Δ*ssaV*::*kan*	This study
T400	TM131 harboring pACYC-gfp, expressing GFP	This study
T387	T434 harboring pACYC-gfp, expressing GFP	This study
T402	T301 harboring pACYC-gfp, expressing GFP	This study
T403	T302 harboring pACYC-gfp, expressing GFP	This study
T404	T303 harboring pACYC-gfp, expressing GFP	This study
T301	SL1344 Δ*ssaV* Δ*amiA*::*kan*	This study
T302	SL1344 Δ*ssaV* Δ*amiC*::*kan*	This study
T303	SL1344 Δ*ssaV* Δ*amiA*::*kan* Δ*amiC*	This study
T410	T303 harboring p*amiA*, expressing AmiA	This study
T411	T303 harboring p*amiC*, expressing AmiC	This study
TM1005	SL1344 Δ*ssaV* Δ*flhA*::*cat*	This study
T436	SL1344 Δ*ssaV* Δ*cheY*::*cat*	This study
T431	SL1344 Δ*ssaV* Δ*flhA*::*kan* Δ*amiA* Δ*amiC*	This study
T435	SL1344 Δ*ssaV* Δ*cheY*::*kan* Δ*amiA* Δ*amiC*	This study
T407	SL1344 Δ*amiA*::*kan* Δ*amiC*	This study
Z349	SL1344 Δ*phoP*::*kan*	Miki & Hardt
T146	SL1344 Δ*ssaV*::*cat* Δ*phoP*::*kan*	This study
T249	SL1344 Δ*invG* Δ*ssaV*::*cat*	[47]
T421	SL1344 Δ*invG*::*kan* Δ*ssaV* Δ*amiA* Δ*amiC*	This study
T440	SL1344 Δ*invG* Δ*ssaV*	This study
T447	T440 harboring pACYC-gfp, expressing GFP	This study
T448	T421 harboring pACYC-gfp, expressing GFP	This study
**Plasmid**	**Genotype**	**Reference**
pMW118	Low-copy number expression vector	NipponGene
p*amiA*	pMW118 containing *amiA*, expressing AmiA	This study
p*amiC*	pMW118 containing *amiC*, expressing AmiC	This study
pACYC-gfp	pACYC184 expressing GFPmut3.1	Lab. Stock

### Construction of complementary plasmid

Complementary plasmids were constructed using DNA fragments containing *amiA* gene or *amiC* gene which amplified by PCR with primer sets: amiA-FW-SacI and amiA-RV-SphI or amiC-FW-SacI and amiC-RV-SphI, and *S*. Tm strains SL1344 chromosomal DNA as template, which were digested with SacI and SphI, and then ligated between the same sites of pMW118, yielding to p*amiA* and p*amiC* respectively. Primers used for construction of complementary plasmid are listed in S1 Table.

### Animal infection experiments

Animal infection experiments were performed in 6 to 12 week old mice as described previously [41, 73]. C57BL/6 mice were maintained at the institute of experiments of animals at School of Pharmacy, Kitasato University or purchased from Japan SLC. To trigger artificial and *S*. Tm virulence independent colitis when required, a dextran sulfate sodium (DSS) was treated prior infection. In brief, sterile-filtered drinking water supplemented with 3.5% DSS (molecular mass 36,000–50,000 g/mol, MP Biomedicals) was provided to the mice *ad libitum* for 7 days. C57BL/6 mice were pretreated with 25 mg streptomycin 24 hours prior to infection. For infection, bacteria were grown for 12 h in LB medium containing 0.3 M NaCl supplemented with appropriate antibiotic(s) under mild aeration (160 rpm), diluted 1:20 and sub-cultured for 4 h in the same medium without supplementation of antibiotics. Bacteria were washed twice with PBS and mice infected with 5×10^7^ CFU *S*. Tm strains by gavage. To determine bacterial population sizes, fecal pellets, cecal content, mLN and spleen were freshly collected in sterile PBS containing 0.5% tergitol, and subjected to bead-beating and plated on MacConkey agar plates (Nissui Pharmaceutical) supplemented with the appropriate antibiotics (50 μg/ml streptomycin; 50 μg/ml kanamycin; 10 μg/ml chloramphenicol). MacConkey medium including bile acids are suitable for determining the total number of *S*. Tm in the gut, even if the *S*. Tm strains display the impaired resistance to deoxycholate. This was demonstrated by comparison of growth on LB or MacConkey medium shows that the impaired resistance of *S*. Tm strains to deoxycholate had no effect on growth MacConkey medium (S10 Fig). Furthermore, bead-beating and plating in the mouse infection experiments in this study are suitable for determining total *S*. Tm CFUs in the gut. This was verified by comparing the bacterial shapes of the long chained *S*. Tm cells with or without bead-beating using microscopy, showing that the long chained cell are sheared into single cells after bead-beating treatment (S11A and S11B Fig). A CI was calculated by dividing the population size of background strains of *S*. Tm by its derivative mutants. Parts of cecal tissue were fixed in 4% formaldehyde (Mildform, Wako Pure Chemical Industries, Ltd.) and embedded in paraffin. Cryosections were prepared and air-dried, and then stained hematoxylin/eosin (H&E). To determine the degree of inflammation, pathological score was monitored as previously described [41], evaluating submucosal edema, polymorphonuclear leukocyte infiltration, goblet cell numbers, and epithelial damage, a total score of 0–13. More than 3 scores are considered as a sign of inflammation. To reduce luminal bile acids which interact with *S*. Tm in the gut, C57BL/6 mice were fed a normal rodent chow supplemented with the bile acid sequestrant cholestimide resin (1.5%, Mitsubishi Tanabe Pharma).

### Lipocalin-2 ELISA

Fecal pellet collected at the indicated time points were homogenized, and diluted in PBS. The resulting dilutions were then analyzed using the mouse lipocalin-2 ELISA duoset (R&D) according to the manufacturer’s instructions.

### *In vitro* bacterial morphology analysis

*S*. Tm strains were grown overnight in LB at 37°C, diluted 1:100 in fresh LB broth, or LB containing 0.5 M NaCl or 1 μg/ml polymyxin B, and grown for 2.5 h. The resulting bacteria were placed on a 1.5% agarose pad, sealed under a glass coverslip, and imaged at 400× using the Zeiss Axiovert A1 microscope.

### Electron microscopy analysis

*S*. Tm strains grown in LB containing 0.5 M NaCl were harvested, resuspended in 2.5% glutaraldehyde in PBS. The samples were post-fixed with 2% osmium tetroxide for 3 h at 4°C, then dehydrated through a series of ethanol concentrations. Specimens were critical-point dried using carbon dioxide. The samples were coated with osmium plasma and examined at 5 kV accelerating voltage in a JSM-6320F SEM.

### Fluorescence microscopy of *S*. Tm in feces

Fecal pellet was suspended gently in PBS. The resulting suspension was placed on a 1.5% agarose pad, sealed under a glass coverslip, and imaged at 400× using the Zeiss Axiovert A1 microscope.

### Motility assay

*S*. Tm strains grown overnight in LB at 37°C, subcultured in fresh LB broth and further grown for 2 h. A 5-μl aliquot at an OD_600_ of 1.0 was placed on 0.3% agar LB plate (for swimming) or 0.5% agar LB plate supplemented with 0.5% glucose (for swarming), and left for 5 min. The plates were incubated at 37°C for 5 h (swimming) or 10 h (swarming).

### Determination of minimum inhibitory concentrations (MICs)

*S*. Tm strains grown to the logarithmic growth phase were diluted to 1×10^6^ CFU per ml with different concentrations of magainin 2 (LKT Laboratories, Inc.) or deoxycholate (Nacalai tesque) in sterile LB broth, and incubated for 15 h at 37°C. A positive control contained no antimicrobials whereas in the negative control, *S*. Tm cells were not present. After incubation, the *A*_595_ values were determined using a microplate reader (Bio-Rad). MICs were determined as the lowest concentrations of antimicrobials that were shown to prevent bacterial growth by more than 50% in comparison with the growth of the positive control.

### EtBr influx assay

Outer membrane permeability was evaluated by EtBr influx assay [53]. *S*. Tm grown to the stationary growth phase was washed with PBS, and diluted to OD_600_ = 0.4/ml in PBS. The resulting bacterial suspensions were mixed with EtBr (24 μM), followed by measuring the fluorescence signal intensity of the EtBr-nucleic acid complex using SpectraMax M5 spectrofluorometer (Molecular Devices) with excitation and emission wavelengths of 544 and 590 nm, respectively.

### Quantification of total bile acids in feces

Fecal pellets were weight, homogenized in ethanol. Total bile acids in feces was extracted by hot ethanol method, and analyzed using Total bile acids-Test wako (FUJIFILM Wako Pure Chemical) according to the manufacturer’s instructions.

### Enumeration of propidium iodide-stained bacteria by deoxycholate killing

*S*. Tm strains were grown overnight in LB at 37°C, diluted 1:100 in fresh LB broth or LB containing 0.5 M NaCl, and grown for further 2.5 h. The bacteria were mixed with 1% deoxycholate in PBS, incubated at 37°C for 20 min. After the incubation, propidium iodide (PI) solution (Dojindo) was added, and incubated further 5 min at room temperature. PI-stained *S*. Tm cells were counted by fluorescence microscopy.

### Statistical analysis

Statistical significance was determined by Mann Whitney *U*-test or Student *t*-test using the software Graphpad Prism. *P* values of less than 0.05 were considered significant (*P < 0.05; ** *P* < 0.01; *** *P* < 0.001).

## Supporting information

S1 Fig *S*. Tm *tatC* or *amiA amiC* mutant strains form chains *in vitro*. (A-C) GFP-expressing *S*. Tm *ssaV*, *ssaV tatC*, *ssaV amiA*, *ssaV amiC*, or *ssaV amiA amiC* strains were grown in LB media. *S*. Tm cells were placed on a 1.5% agarose pad, sealed under a glass coverslip, and observed by fluorescence microscopy. (A) Representative fluorescence microscopy images of GFP-expressing *S*. Tm strains grown in LB media (400x). Scale bar, 20 μm. (B) Quantitative analyses of the experiments. At least three independent experiments were performed. Bars represent mean ± SD from four experiments. ns, not significant; *P < 0.05; **P < 0.01; ***P < 0.001; unpaired Student’s t-test. (C) Proportion of chains. Bars represent mean ± SD from five experiments. *P < 0.05; ***P < 0.001; unpaired Student’s t-test. (D) Representative scanning electronic microscopy (SEM) images of *S*. Tm strains grown in LB media (10,000x). Scale bar, 1 μm.
(EPS)

S2 Fig Introduction of *amiC* gene into *S*. Tm *ssaV amiC* mutant restores the competitive fitness in the gut. Streptomycin-treated C57BL/6 mice (n = 8 per group) were infected for 6 days with 1:1 mixture (total 5×10^7^ CFU intragastrically) of *S*. Tm *ssaV* and *ssaV amiC* or *ssaV* and *ssaV amiC* p*amiC* strains. CIs of *S*. Tm loads in feces at 1 and 6 dpi were determined. Bar indicates median. ns, not significant (*P* ≥ 0.05); ***P < 0.001; Mann-Whitney U test.
(EPS)

S3 Fig *S*. Tm AmiC, but not AmiA, is involved in the competitive fitness in the gut. Streptomycin-treated C57BL/6 mice (n = 6 per group) were infected for 5 days with 1:1 mixture (total 5×10^7^ CFU intragastrically) of *S*. Tm *ssaV* and *ssaV amiA amiC* p*amiA* or *ssaV* and *ssaV amiA amiC* p*amiC* strains. CIs of *S*. Tm loads in feces at 1 and 5 dpi were determined. Bar indicates median. ns, not significant (*P* ≥ 0.05); **P < 0.01; Mann-Whitney U test.
(EPS)

S4 Fig Impaired colonization of *S*. Tm *ssaV amiA amiC* mutant strain. Streptomycin-treated C57BL/6 mice (n = 11 per group) were infected for 8 days with 5×10^7^ CFU intragastrically of *S*. Tm strains *ssaV* or *ssaV amiA amiC*. (A) *S*. Tm loads in feces at 1, 3, and 8 dpi. (B) *S*. Tm loads in cecum at 8 dpi. (C) Representative H&E stained cecal section (100x). Scale bar, 20 μm. lu, lumen; mu, mucosa; s.e., submucosal edema. (D) Cecal pathological score in H&E-stained cecal tissue section. Bar indicates median. ns, not significant; *P < 0.05; **P < 0.01; ***P < 0.001; Mann-Whitney U test.
(EPS)

S5 Fig *S*. Tm *invG ssaV amiA amiC* mutant strains form chains *in vitro*. GFP-expressing *S*. Tm *invG ssaV* and *invG ssaV amiA amiC* strains grown in LB media were placed on a 1.5% agarose pad, sealed under a glass coverslip, and observed by fluorescence microscopy. Representative fluorescence microscopy images of GFP-expressing *S*. Tm strains grown in LB media (400x). Arrow indicates chain form of *S*. Tm. Scale bar, 20 μm.
(EPS)

S6 Fig Effect of DSS treatment on body weight of mice. C57BL/6 mice (n = 7) were exposed to 3.5% DSS *ad libitum* for 7 days, whereas control mice (n = 7) were given sterile water. Body weight was measured at 0, 3, 5 and 7 days after DSS treatment. Data points represent means ± standard deviations. ns, not significant; *P < 0.05; ***P < 0.001; unpaired Student’s t-test.
(EPS)

S7 Fig Involvement of chemotaxis in AmiA- and AmiC-dependent competitive fitness. (A and B) *S*. Tm strains were plated on LB with 0.5% agar (swarming), and incubated at 37°C for 10 hours. (C and D) Streptomycin-treated C57BL/6 mice (n = 8) were infected for 4 days with 1:1 mixture (total 5×10^7^ CFU intragastrically) of *S*. Tm *ssaV cheY* and *ssaV cheY amiA amiC* mutants. (C) CIs of *S*. Tm loads in feces at 1 and 4 dpi were determined. (D) Fecal lipocalin-2 ELISA. Bar indicates median. ***P < 0.001; Mann-Whitney U test.
(EPS)

S8 Fig Feeding with the bile sequestrant cholestimide resin enhances excretion of bile acids in feces. C57BL/6 mice (n = 3 per group) were fed with chow containing cholestimide resin or control chow for 7 days. (A) Experimental strategy. (B) Concentrations of total bile acids in feces. Bar indicates median. *P < 0.05; unpaired Student’s t-test.
(EPS)

S9 Fig Polymyxin B imposes AmiA- and AmiC-dependent cell division on *S*. Tm *in vitro*. GFP-expressing *S*. Tm *ssaV* or *ssaV amiA amiC* strains grown in LB supplemented with 1 μg/m polymyxin B were observed by fluorescence microscopy. Proportion of chains. Four independent experiments were performed. Bars represent mean ± SD. **P < 0.01; unpaired Student’s t-test.
(EPS)

S10 Fig *S*. Tm *tatC* or *amiA amiC* mutant strains can grow on MacConkey agar plate. *S*. Tm *ssaV*, *ssaV tatC*, or *ssaV amiA amiC* strains were grown in early stationary phase (*A*_600_ = 1.0). A 5-μl aliquot of the *S*. Tm culture at an OD_600_ of 0.5 was spotted on LB agar plate or MacConkey agar plate, and the plates were incubated in a 37°C incubator for 20 h. Representative of three independent experiments was shown.
(EPS)

S11 Fig Bead-beating can break down the chains of *S*. Tm *amiA amiC* mutant cells. GFP-expressing *S*. Tm *ssaV* or *ssaV amiA amiC* strains were grown in LB plus 0.5 M NaCl, and subsequently *S*. Tm cells were collected, followed by bead-beating for 2 min. The resulting *S*. Tm cells were observed by fluorescence microscopy. (A) Representative fluorescence microscopy images of GFP-expressing *S*. Tm (400x). Scale bar, 20 μm. (B) Quantitative analyses of the experiments. Six independent experiments were performed. Bars represent mean ± SD. ns, not significant; *P < 0.05; unpaired Student’s t-test.
(EPS)

S1 Table Primers used in this study. (XLSX)
